# The effect of intravenous unit-dose tranexamic acid on visible and hidden blood loss in posterior lumbar interbody fusion: a randomized clinical trial

**DOI:** 10.1038/s41598-022-27307-3

**Published:** 2023-03-22

**Authors:** Shenshen Hao, Hongke Li, Shuai Liu, Saike Meng, Xiaopeng Zhang, Linfei Wang, Hongjie Yang, Liyan Zhang, Shengli Dong

**Affiliations:** Department of Spine and Bone Oncology, General Hospital of Pingmei Shenma Medical Group, Pingdingshan, 467000 Henan China

**Keywords:** Diseases, Medical research, Neurology

## Abstract

There are few reports of intravenous unit-dose tranexamic acid (TXA) on the relationship between visible blood loss (VBL) and hidden blood loss (HBL) in posterior lumbar interbody fusion (PLIF). Therefore, the objective of this randomized, prospective, double-blind, single center study was to investigate the effect of intravenous unit-dose TXA on VBL and HBL in patients who underwent PLIF. Among 100 patients, 11 were excluded due to failue to comply with the study, 1 was excluded due to non-conpliance with the study, and 88 were eligible for inclusion in the study. 46 patients who treated with PLIF received unit-dose of TXA (1 g/100 mL) intravenously 15 min before skin incision after general anesthesia (observation group) and 42 patients were given 100 mL of normal saline (control group). The operation time, intraoperative blood loss, postoperative drainage, VBL, HBL, blood transfusion rate, and adverse events were recorded in the two groups. Besides, activated partial prothrombin time (APTT), prothrombin time (PT), thrombin time (TT), fibrinogen (FIB), platelets (PLT), red blood cells (RBC), hemoglobin (HB), hematocrit (HCT), C-reactive protein (CRP), erythrocyte sedimentation rate (ESR) on the 1st postoperative day; and RBC, HB, HCT, CRP, ESR on the 4th postoperative day were recorded. All 88 patients successfully completed the operation, the incision healed well, and there was no deep vein thrombosis of the lower extremity after operation. The intraoperative blood loss, postoperative drainage, VBL, HBL, and blood transfusion rate in the observation group were lower than those in the control group, and the differences were statistically significant (p < 0.05). There was no significant difference in operation time between the two groups (p > 0.05). There was no significant difference in postoperative APTT, PT, TT, FIB, PLT, RBC, HB, HCT, CRP and ESR between the two groups (p > 0.05). Intravenous unit-dose TXA is safe and feasible in PLIF, and it can effectively reduce perioperative VBL and HBL.

## Introduction

Lumbar diseases such as lumbar disc herniation, lumbar spinal stenosis and lumbar spondylolisthesis are common diseases of people. With the aging of the population, its incidence also increases^1–3^. Posterior lumbar interbody fusion (PLIF) is currently a classic surgical method for the treatment of these diseases^4^. However, PLIF requires extensive soft tissue dissection, decompression, and interbody fusion, resulting in massive perioperative blood loss^5^. Therefore, blood transfusion is often required, and it can cause some complications, such as hemolysis and infectious diseases^6^. Generally speaking, only visible blood loss (VBL) is assessed when assessing bleeding during perioperative period, which includes intraoperative bleeding and postoperative drainage. Hidden blood loss (HBL) due to haemolysis and residual blood in the dead space is often overlooked, so true blood loss is always underestimated^7^. A large amount of HBL is an important cause of postoperative anemia^8^. The study found that in two-segment PLIF, perioperative HBL accounted for 37% to 44% of the total blood loss^9^. It can be seen that HBL is a large part of the total blood loss in PLIF^10^. Large amounts of HBL may impair postoperative recovery^11^. Therefore, reducing HBL and VBL are equally important.

Tranexamic acid (TXA) is a synthetic antifibrinolytic agent that inhibits plasminogen, fibrinolysis and tissue plasminogen activator binding by competing for lysine binding sites. Thereby delaying fibrinolysis and blood clot degradation, TXA achieves the purpose of reducing intraoperative bleeding^12^. Studies have shown that the application of TXA during PLIF can reduce the intraoperative bleeding, postoperative drainage, and blood transfusion rate, and does not increase the risk of deep venous thrombosis (DVT)^13,14^. It can be seen that the use of TXA has a positive effect on the blood management of patients undergoing PLIF^15^. However, there are few reports on the relationship between VBL and HBL in PLIF with intravenous unit-dose TXA.

There are various schemes for perioperative application of TXA, such as single preoperative intravenous administration, continuous intravenous infusion, topical administration, and intravenous combined topical administration^16,17^. The advantage of intravenous TXA, not interfering with other intraoperative intravenous drugs, is that it acts on the whole body to inhibit the fibrinolytic system activated by surgery to effectively reduce VBL and HBL^18^. With the activation of the coagulation system, the fibrinolytic system is also activated, and surgery results in a transient cascade of activation of fibrinolysis^19^. Studies have shown that preoperative intravenous TXA is far more effective in inhibiting blood clot breakdown than after fibrinolytic system activation^20^. Therefore, preoperative administration of antifibrinolytic drugs such as TXA is the key to reduce intraoperative bleeding^21^. Studies have found that about 15 min after intravenous of TXA could pass through the body's physiological barrier, enter and accumulate in surgical and trauma areas^22^. Therefore, intravenous application of TXA 15 min before surgery can make TXA play a role in inhibiting fibrinolysis and reduce bleeding^23^. Therefore, intravenous TXA 15 min before skin incision after general anesthesia was one of the main recommendation in the 2019 Chinese Expert Consensus^24^. Therefore, this randomized clinical trail aims to analyze the application effect of unit-dose TXA on VBL and HBL in PLIF.

## Materials and methods

### General information

**Figure 1:**
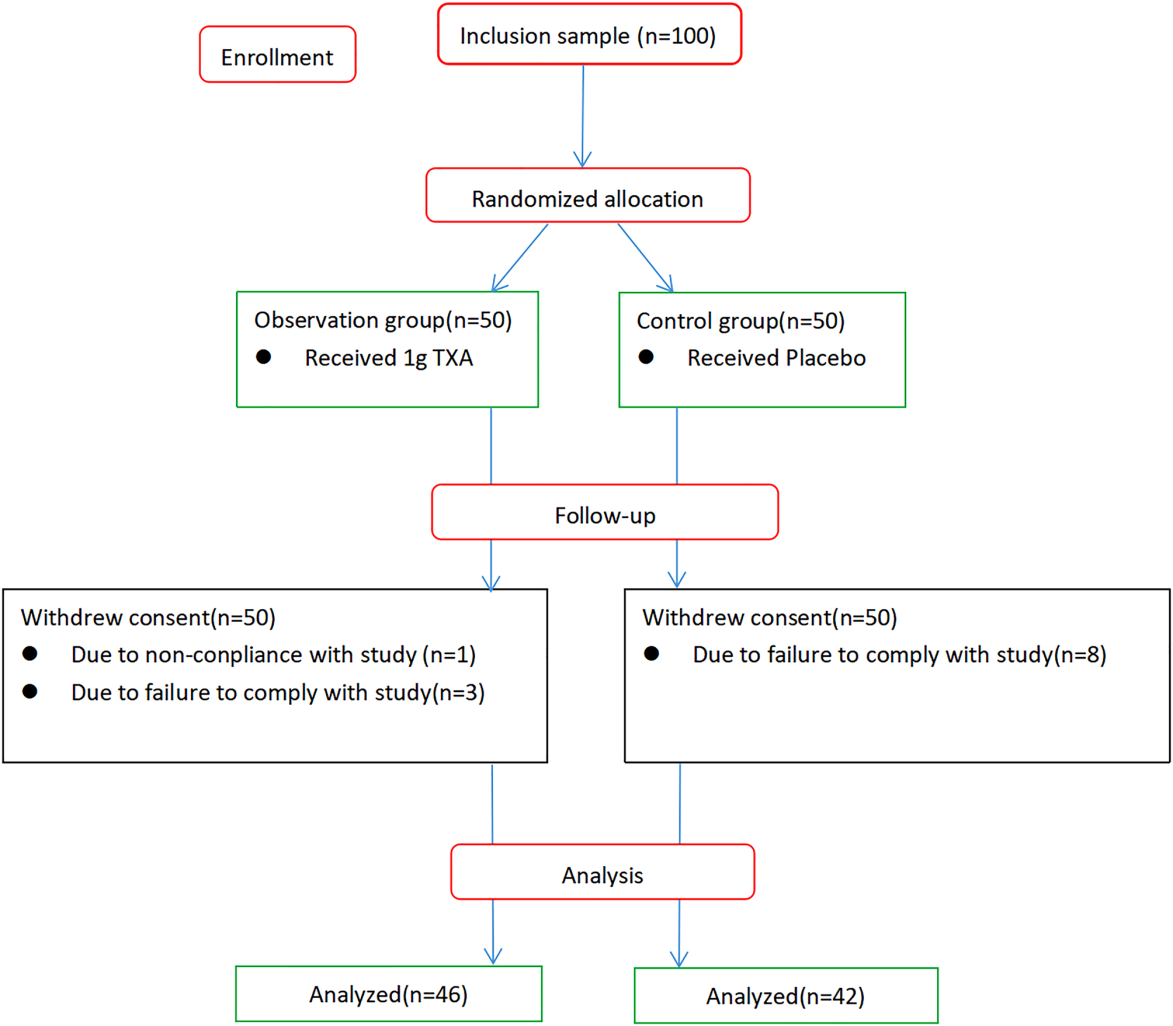
Disposition of the study participants.

This was a double blind randomized placebo control study and was approved by the Ethics Committee of the General Hospital of Pingmei Shenma Medical Group, and the reference number is 2021004. The study performed in the Department of Spine of General Hospital of Pingmei Shenma Medical Group from 2020.11 to 2022.3. The initial plan of the study was to include 100 patients who were equally randomly assigned to been given either TXA as the observation group or 0.9% normal saline as the control group using odd and even numbers table. The identical drug information of TXA and placebo was covered, and the surgeon and anesthesiologist did not know the contents of the drug bags before data statistical analyzed. The inclusion criteria of the study included: those who were diagnosed with lumbar disc herniation, lumbar spinal stenosis and lumbar spondylolisthesis and required PLIF; those who underwent surgery for the first time; those who could tolerate general anesthesia. The exclusion criteria of the study included: patients with fracture injury; patients with preoperative DVT of lower extremities; patients with intraoperative cerebrospinal fluid leakage or dural damage. 88 patients out of the initial 100 patients were eligible for inclusion in the study: 46 patients were in the observation group, with three patients excluded due to failure to comply with the study and one patient excluded due to non-conplicance with the study^25^, while 42 patients were in the control group with eight patients excluded due to failure to comply with the study (Fig. 1).

The total 88 cases included 46 males and 42 females, aged 34–88 years, with an average age of (59.35 ± 10.52) years. The gender, age, body mass index (BMI), disease type, surgical fusion segment, and preoperative activated partial prothrombin time (APTT), prothrombin time (PT), thrombin time (TT), fibrinogen (FIB), platelets (PLT), red blood cells (RBC), hemoglobin (HB), hematocrit (HCT), C-reactive protein (CRP), erythrocyte sedimentation rate (ESR) were served as general data (Table 1).

**Table 1:** The general data of the two groups.

	Observation group (n = 46)	Control group (n = 42)	t/z/χ^2^	P
**Sex, n (%)**			1.119	0.290
Male	20 (43)	23 (55)		
Female	26 (57)	19 (45)		
BMI, kg/m^2^	24.15 ± 2.59	25.34 ± 3.15	−1.949	0.055
Age, year	60.09 ± 10.05	58.55 ± 11.07	0.684	0.496
PT, s	11.2 [11.0; 11.7]	11.0 [10.6; 11.5]	−1.865	0.062
APTT, s	30.90 ± 2.54	30.94 ± 3.36	−0.056	0.956
FIB, g/L	2.83 [2.48; 3.07]	2.64 [2.43; 3.02]	−0.940	0.347
TT, s	14.50 [13.90; 15.30]	14.98 [14.23; 15.81]	−1.471	0.141
RBC, 10^12^/L	4.29 ± 0.45	4.42 ± 0.40	−1.488	0.140
HB, g/L	136.52 ± 14.29	137.62 ± 14.14	−0.362	0.719
HCT, L/L	0.40 ± 0.04	0.41 ± 0.04	−0.878	0.382
PLT, 10^9^/L	213.5 [187.0; 244.0]	205.5 [175.0; 261.0]	−0.409	0.682
CRP, mg/L	0.00 [0.00; 0.99]	0.11 [0.00; 1.99]	−0.946	0.344
ESR, mm/h	16.0 [8.0; 26.0]	12.0 [6.0; 21.0]	−0.995	0.320
**Disease type, n (%)**			1.885	0.390
LDH	5 (11)	8 (19)		
LSS	28 (61)	20 (48)		
LS	13 (28)	14 (33)		
**Surgical fusion segment, n (%)**			1.761	0.415
One	20 (43)	24 (57)		
Two	20 (43)	13 (31)		
Three	6 (14)	5 (12)		

*n *number, *HB *hemoglobin, *PLT *platelet, *APTT *activated partial thromboplastin time, *PT *prothrombin time, *TT *thrombin time, *FIB *fibrinogen, *BMI *body mass index, *LSS *lumbar spinal stenosis, *LDH *lumbar disc herniation, *LS *lumbar spondylolisthesis.

### Treatment methods

88 patients were all under general anesthesia, prone position. The observation group received a unit-dose of TXA (Containing 100 mL normal saline and 1 g TXA, Drug batch No. H20031101; Chongqing Lummy Pharmaceutical Co., Ltd) intravenously 15 min before skin incision after general anesthesia. The control group was given 100 mL of 0.9% normal saline in the same way. The two groups had the same operation, that was, the standard PLIF, including bilateral fenestration and subtraction. A longitudinal incision was made centered on the diseased vertebral segment. The skin was separated layer by layer, subcutaneously to the spinous process, the bilateral paraspinal muscles were stripped, the spinous process, lamina, and articular process were exposed, and then the pedicle screw was placed, fenestration decompression, intervertebral discectomy, and bone graft fusion were performed. The incision was sutured layer by layer, and two negative pressure drainage tubes were placed. When the patients returned to the ward, they were given bed rest, routine use of antibiotics to prevent infection, and application of hormones, dehydration, anticoagulation and pain relief. Perioperative blood transfusion was red blood cell, and the standard of blood transfusion was that the hemoglobin level was lower than 70 g/L. The vital signs, surgical incision, sensation and movement of both lower extremities were observed. The condition for removing the drainage tube was when the drainage volume was less than 50 mL/24 h. When a patient suddenly presented with lower extremity pain, bilateral skin temperature, or unequal circumference, urgent ultrasonography was performed to determine whether there was DVT of the lower extremity.

### Observation indicators

The operation time, intraoperative blood loss, postoperative drainage, VBL, HBL, blood transfusion rate, and adverse events were recorded in the two groups. APTT, PT, TT, FIB, PLT, RBC, HB, HCT, CRP, ESR on the 1st postoperative day; and RBC, HB, HCT, CRP, ESR on the 4th postoperative day were also recorded. Adverse events included poor postoperative wound healing and postoperative DVT.

The measurement of intraoperative blood loss was as follows. It included the amount of drainage in the intraoperative drainage bottle minus the amount of saline used to flush the incision during the operation, plus the net added weight of the cotton used for hemostasis during the operation. The postoperative drainage was calculated as the total drainage volume in the postoperative drainage tubes. The calculation of HBL was obtained according to the literature method^26^: VBL = intraoperative blood loss + postoperative drainage volume; HBL = calculated total blood loss + blood transfusion volume − VBL. According to Gross formula^27^ and Nadel formula^28^, calculated total blood loss (L) = patient′s blood volume (PBV) * (preoperative HCT − postoperative HCT)/mean HCT, where mean HCT = (preoperative HCT + postoperative HCT)/2, PBV (L) = k1 × height (m)^3^ + k2 × weight (kg) + k3 (male k1 = 0.366 9, k2 = 0.032 19, k3 = 0.604 1; female k1 = 0.356 1, k2 = 0.033 08, k3 = 0.183 3).

### Statistical methods

Data analysis was performed using SPSS 22.0 statistical software. Measurement data conforming to normal distribution were expressed as mean ± standard deviation, and t-test was used for comparison between groups. Non-normally distributed measurement data were represented by M[P25; P75], and the comparison between groups was performed using the Mann–Whitney U nonparametric test. The enumeration data were described in the form of the number of cases (percentage), and the chi-square test was used for comparison between groups. P < 0.05 was considered statistically significant.

### Ethics approval and consent to participate

This study had been performed in accordance with the Declaration of Helsinki, and was approved by the Ethics Committee of the General Hospital of Pingmei Shenma Medical Group, and the reference number is 2021004. All authors confirmed that informed consent was obtained from all subjects.

## Results

There was no significant difference in the general data of two groups before surgery (p > 0.05) (Table 1).

All 88 patients successfully completed the operation, and no adverse events occurred after operation. There was no significant difference in operation time between the two groups (p > 0.05). The intraoperative blood loss, postoperative drainage, VBL, HBL, and blood transfusion rate in the observation group were lower than those in the control group, and the differences were statistically significant (p < 0.05). There was no significant difference in postoperative APTT, PT, TT, FIB, PLT, RBC, HB, HCT, CRP and ESR between the two groups (p > 0.05) (Table 2).

**Table 2:** The observation indicators of the two groups.

	Observation group (n = 46)	Control group (n = 42)	t/z/χ^2^	P
Operation time, min	175.0 [150.0; 215.0]	180.0 [160.0; 215.0]	−0.435	0.664
Intraoperative blood loss, mL	300.0 [300.0; 500.0]	425.0 [300.0; 600.0]	−2.274	0.023
Postoperative drainage, mL	220.0 [200.0; 270.0]	330.0 [310.0; 400.0]	−6.357	< 0.001
VBL, mL	580.0 [500.0; 770.0]	810.0 [620.0; 1040.0]	−4.011	< 0.001
HBL, mL	353.12 [235.71; 474.10]	524.37 [387.60; 640.55]	−3.141	0.002
**Blood transfusion rate, n (%)**			12.623	< 0.001
Received	8 (17)	12 (29)		
Not received	38 (83)	30 (71)		
PT(post-1d), s	12.3[11.6; 12.9]	12.2 [11.8; 12.9]	−0.268	0.789
APTT(post-1d), s	28.32 ± 2.02	28.57 ± 2.61	−0.512	0.610
FIB(post-1d), g/L	2.93[2.62; 3.15]	2.76 [2.56; 3.12]	−1.14	0.254
TT(post-1d), s	14.28 ± 0.97	14.40 ± 0.91	−0.602	0.549
RBC(post-1d), 10^12^/L	3.72 ± 0.43	3.66 ± 0.34	0.651	0.517
HB(post-1d), g/L	117.39 ± 14.42	115.24 ± 12.18	0.753	0.453
HCT(post-1d), L/L	0.34 ± 0.04	0.34 ± 0.03	0.865	0.389
PLT(post-1d), 10^9^/L	181.0 [154.0; 230.0]	181.0 [146.0; 214.0]	−0.693	0.488
CRP(post-1d), mg/L	17.56 [10.22; 31.16]	17.08 [12.93; 27.82]	−0.159	0.874
ESR(post-1d), mm/h	7.5 [3.0; 14.0]	4.0 [2.0; 10.0]	−1.876	0.061
RBC(post-4d), 10^12^/L	3.54 ± 0.48	3.43 ± 0.38	1.112	0.269
HB(post-4d), g/L	112.15 ± 15.54	108.60 ± 13.06	1.156	0.251
HCT(post-4d), L/L	0.33 ± 0.05	0.31 ± 0.04	1.772	0.080
PLT(post-4d), 10^9^/L	194.0 [156.0; 227.0]	187.0 [140.0; 236.0]	−0.898	0.369
CRP(post-4d), mg/L	7.37 [3.66; 16.01]	14.81 [3.35; 33.92]	−1.429	0.153
ESR(post-4d), mm/h	31.0 [13.0; 43.0]	26.5 [11.0; 39.0]	−0.405	0.685

*n *number, *HB *hemoglobin, *PLT *platelet, *APTT *activated partial thromboplastin time, *PT* prothrombin time, *TT *thrombin time, *FIB *fibrinogen, *post-1d *the 1st day after surgery, *post-4d *the 4th day after surgery.

## Discussion

The main links of bleeding caused by PLIF include these steps, such as bleeding from the paraspinal muscle tissue during exposure, bleeding from the cancellous bone surface during laminectomy and decompression, and bleeding from the spinal venous plexus during intraspinal operation and posterior incision drainage results in blood loss^18^. Under normal circumstances, there is a dynamic balance between coagulation and anticoagulation, fibrinolysis and antifibrinolysis in the human body^20^. When the blood vessels in the tissue are injured during surgery, the coagulation system and fibrinolysis are activated almost simultaneously^29^. The coagulation system is activated to generate blood clots, and fibrin in the blood clots can adsorb plasminogen and activators to form a large amount of plasmin, which dissolves the clots and causes further bleeding^30^. Antifibrinolytic drugs such as TXA can reversibly combine with plasminogen, preventing the activation of plasminogen to plasmin, thereby producing hemostasis^29^.

VBL generally refers to intraoperative bleeding and postoperative drainage. The results of our study showed that the intraoperative blood loss, postoperative drainage, and VBL of the observation group were significantly smaller than those of the control group. It shows that the intravenous unit-dose of TXA can effectively reduce the VBL of PLIF. Theoretically, intraoperative hemorrhage will directly affect the clarity of the surgical field, which may prolong the operation time. However, our study found no significant difference in operative time between the two groups. We think there are two possible reasons for this result. First, our sample size is relatively small, which may affect the results. Second, it is possible that the surgeon's skill is good, so that the influence of intraoperative bleeding becomes less obvious.

The HBL phenomenon was first discovered by Sehat et al.^31^. They studied patients with rheumatoid arthritis who underwent total knee arthroplasty and found that the patients had varying degrees of decreased HB after surgery. HBL refers to a special form of blood loss that cannot be seen clinically and is difficult to directly estimate, but it is very easy to ignore^32^. Up to now, the specific mechanisms and causes of HBL are not fully understood. The possible explanations are as follows: the trauma leads to the infiltration of blood into the interstitial space^8^; the increased permeability of the cell membrane of some tissues in the trauma environment leads to hemolysis after the swelling and rupture^33–35^; the increase in the fibrinolytic system during stress tissue plasminogen activator, which increases the potential for bleeding^36^. Especially for elderly patients with PLIF, the loose back muscle space provides a larger space for HBL, and the existence of an internal fixation system provides a potential space for HBL^32^. In addition, organ dysfunction, coupled with the under-recognized seriousness of HBL, makes this problem a major threat to patients' perioperative life safety^37^. Our study showed that intravenous unit-dose of TXA can significantly reduce HBL in PLIF. We believe that its mechanism may be as follows: TXA can reduce the small blood vessel bleeding and muscle tissue oozing that are difficult to find during surgery^30^; promote coagulation and maintain vascular permeability to reduce blood entering the interstitial space^37^.

Generally speaking, the application of TXA reduces the intraoperative blood loss and postoperative drainage of patients, and also reduces the incidence of postoperative anemia^23^. However, our study did not yield similar results. Our study suggested that the related anemia indexes (including RBC, HB, HCT) were similar between the two groups. For this result, we believe that the possible reason is related to blood transfusion. Because the transfusion rate in the control group was greater than that in the observation group, postoperative anemia was improved. Meanwhile, we also observed changes in CRP and ESR. They are indicators that reflect the inflammatory response after PLIF, which may show the body's inflammatory response to some extent. However, there are few observations and inconsistent conclusions^23,30^. As there is a certain correlation between fibrinolysis system and body inflammation, TXA may play an indirect role in inhibiting inflammatory response by inhibiting fibrinolysis^38^. At present, the research on the anti-inflammatory effect of TXA mainly focuses on the field of joint replacement^39–41^. For example, TXA could reduce perioperative inflammatory indicators in knee replacement^42^. Our study suggests that intravenous unit-dose of TXA has no significant effect on CRP and ESR of PLIF. This also indicates that TXA may not interfere significantly with the inflammatory response to PLIF.

Although, the hemostatic mechanism of TXA relies on inhibition of the fibrinolytic system rather than promotion of coagulation, and theoretically does not increase the risk of thrombosis. The potential thrombosis risk of TXA is also a concern of clinicians while using TXA for hemostasis^43^. Seol et al.^44^ reported that perioperative use of TXA was not associated with thrombosis, and that the site of action of TXA was mainly in surgical wounds rather than peripheral veins. However, patients still have a high risk of thrombosis after PLIF, because they need to stay in bed for a long time and their activities are significantly reduced. Therefore, the safety of intravenous unit-dose of TXA in PLIF must be considered. Our study showed that the operations in both groups were completed safely, with good postoperative healing and no adverse events. Meanwhile, there was no significant difference in the related coagulation indexes (including APTT, PT, TT, FIB, and PLT) between the two groups. It shows that the intravenous application of unit-dose TXA has good safety in PLIF. However, it is worth noting that there are currently no high-quality studies demonstrating the safety of TXA in patients with a history of thromboembolism^45^. Therefore, patients with a history of thromboembolism should be vigilant and not recommended.

The most commonly used method of TXA in spinal surgery is intravenous^12^. Currently, there are no guidelines to clarify the dose of TXA used in spinal surgery^13^. There are often two extreme cases in the process of applying TXA^13^. On the one hand, the dose is insufficient, resulting in no significant reduction in bleeding. Farrokhi et al.^46^ believed that prophylactic low-dose TXA had no significant effect on intraoperative blood loss and blood transfusion demand of patients undergoing spinal fixation surgery. On the other hand, the use of excessive doses may increase the risk of related complications. Our study used a preoperative intravenous unit-dose of TXA, which is a safe and effective method. Moreover, it also has some advantages. On the one hand, compared with the method of preoperative application and intraoperative continuous infusion of TXA, the application of a unit-dose of TXA is not only easier to operate^47^, but also has no interference with intraoperative fluid, such as avoiding drug interactions^30^. On the other hand, compared with the method of topical application of TXA, the application of a unit-dose of TXA can not only avoid the unmaintained local TXA concentration due to postoperative drainage, but also avoid the risk of Staphylococcus aureus infection^48^ and Epilepsy risk in case of dural rupture^49^.

In conclusion, intravenous unit-dose of TXA is safe and feasible in PLIF, it can reduce perioperative VBL and HBL, and it does not affect coagulation function and inflammatory response. However, this study also has shortcomings. This study is a small sample, single centre randomized clinical study.

## Data Availability

The datasets used and analyzed during the current study are available from the corresponding author upon reasonable request.

## Abbreviations

TXA: Tranexamic acid
PLIF: Posterior lumbar interbody fusion
VBL: Visible blood loss
HBL: Hidden blood loss
PLT: Platelet
APTT: Activated partial thromboplastin time
PT: Prothrombin time
TT: Thrombin time
FIB: Fibrinogen
RBC: Red blood cell
HB: Hemoglobin
HCT: Hematocrit
CRP: C-reactive protein
ESR: Erythrocyte sedimentation rate
BMI: Body mass index
LSS: Lumbar spinal stenosis
LDH: Lumbar disc herniation
LS: Lumbar spondylolisthesis
DVT: Deep venous thrombosis

## References

[1] Abi-Hanna D (2018). Lumbar disk arthroplasty for degenerative disk disease: Literature review. World Neurosurg..

[2] Gao T (2017). Correlation between facet tropism and lumbar degenerative disease: A retrospective analysis. BMC Musculoskelet. Disord..

[3] Elsarrag M (2019). Enhanced recovery after spine surgery: A systematic review. Neurosurg. Focus.

[4] Mobbs RJ (2015). Lumbar interbody fusion: Techniques, indications and comparison of interbody fusion options including PLIF, TLIF, MI-TLIF, OLIF/ATP, LLIF and ALIF. J. Spine Surg..

[5] Machado GC (2017). Trends, complications, and costs for hospital admission and surgery for lumbar spinal stenosis. Spine (Phila Pa 1976).

[6] Vamvakas EC, Blajchman MA (2009). Transfusion-related mortality: The ongoing risks of allogeneic blood transfusion and the available strategies for their prevention. Blood.

[7] Elgafy H (2010). Blood loss in major spine surgery: Are there effective measures to decrease massive hemorrhage in major spine fusion surgery?. Spine (Phila Pa 1976).

[8] Zhu X-R (2019). Effect of repeated intravenous application of tranexamic acid on blood loss in posterior lumbar interbody fusion. Orthop. J. Chin..

[9] Zhang Y (2016). Comparison of blood loss of two different laminectomy decompression in treatment of patients with lumbar degenerative disc disease. Acad. J. Chin. PLA Med. Sch..

[10] Lei F (2020). Hidden blood loss and the risk factors after posterior lumbar fusion surgery: A retrospective study. Medicine (Baltimore).

[11] Xu D (2017). The further exploration of hidden blood loss in posterior lumbar fusion surgery. Orthop. Traumatol. Surg. Res..

[12] Cheriyan T (2015). Efficacy of tranexamic acid on surgical bleeding in spine surgery: A meta-analysis. Spine J..

[13] Qiao X (2021). Meta-analysis of the safety and effectiveness of tranexamic acid in the posterior lumbar interbody fusion technique. Chin. J. Spine Spinal Cord.

[14] Elwatidy, S.*, et al.**Efficacy and Safety of Prophylactic Large Dose of Tranexamic Acid in Spine Surgery: A Prospective, Randomized, Double-Blind, Placebo-Controlled Study*. 1528–1159. **(electronic).** 10.1097/BRS.0b013e318188b9c5 19011538

[15] Yang M-K (2019). Role of tranexamic acid in perioperative blood management of elderly patients with lumbar spinal stenosis. J. Spinal Surg..

[16] Zhang Y (2018). Effect of intravenous tranexamic acid on perioperative hidden blood loss in percutaneous pedicle screw fixation for thoracolumbar fractures. Chin. J. Orthop. Trauma.

[17] Chang L (2017). A clinical study on the topical application of tranexamic acid + gelatin sponge in lumbar surgery. Chin. J. Bone Jt..

[18] Chen J (2015). Efficacy and safety of intravenous injection combined with topical application of tranexamic acid in reducing perioperative bleeding of patients with lumbar spine surgery. Chin. J. Biochem. Pharm..

[19] Levy JH (2018). Antifibrinolytic therapy and perioperative considerations. Anesthesiology.

[20] Fang H (2017). Research progress in the application of tranexamic acid in the lumbar vertebra surgery. Chin. Med. Herald.

[21] Lin C (2016). Is combined topical with intravenous tranexamic acid superior than topical, intravenous tranexamic acid alone and control groups for blood loss controlling after total knee arthroplasty: A meta-analysis. Medicine.

[22] Hsieh PW (2013). Co-drug strategy for promoting skin targeting and minimizing the transdermal diffusion of hydroquinone and tranexamic acid. Curr. Med. Chem..

[23] Zhao P (2019). The application of tranexamic acid in lumbar fusion and internal fixation in the elderly. Guangdong Med. J..

[24] Zhou Z (2019). Expert consensus on the application of tranexamic acid and anticoagulant for the enhanced recovery after orthopedic surgery in China. Chin. J. Bone Jt. Surg..

[25] Hao S (2022). Endoscopy-aided retrieval of broken drainage tube after lumbar spine surgery. BMC Surg..

[26] Sehat KR (2004). Hidden blood loss following hip and knee arthroplasty. Correct management of blood loss should take hidden loss into account. J. Bone Jt. Surg..

[27] Gross JB (1983). Estimating allowable blood loss: corrected for dilution. Anesthesiology.

[28] Nadler SB (1962). Prediction of blood volume in normal human adults. Surgery.

[29] Zhong D-G (2018). Tranexamic acid reduces perioperative blood loss in thoracolumbar posterior fusion: A meta-analysis. J. Clin. Rehabil. Tissue Eng. Res..

[30] Yuan J (2021). Effect of adequate amount of tranexamic acid before operation on blood loss and safety in posterior lumbar fusion with multiple segments. Chin. J. Blood Transfus..

[31] Sehat KR (2000). How much blood is really lost in total knee arthroplasty? Correct blood loss management should take hidden loss into account. Knee.

[32] Ban Z-T, Liu R-Z (2018). Progress on hidden blood loss after lumbar interbody fusion. J. Chin. Orthop. Traumatol..

[33] Wen L (2018). Hidden blood loss in anterior cervical fusion surgery: An analysis of risk factors. World Neurosurg..

[34] Yin H (2019). Analysis of related risk factors of hidden blood loss after anterior cervical fusion. Orthopade.

[35] Bai B (2019). Prediction of hidden blood loss during posterior spinal surgery. Chin. Med. Sci. J..

[36] Li Q, Zeng J-C (2015). A clinical study on the application of tranexamic acid in posterior lumbar fusion. West Chin. Med. J..

[37] Yang X (2020). Efficacy and safety of blood loss with different dose of tranexamic acid in lumbar stenosis surgery for elderly patients. Chin. J. Spine Spinal Cord.

[38] Busuttil SJ (2004). A central role for plasminogen in the inflammatory response to biomaterials. J. Thromb. Haemost..

[39] Xu H (2020). Research progress on the anti-inflammatory effects of dexamethasone and tranexamic acid in total joint arthroplasty. Chin. J. Bone Jt..

[40] Xie J (2016). Multiple boluses of intravenous tranexamic acid to reduce hidden blood loss after primary total knee arthroplasty without tourniquet: A randomized clinical trial. J. Arthroplasty.

[41] Lei Y (2017). The efficacy and safety of multiple-dose intravenous tranexamic acid on blood loss following total knee arthroplasty: a randomized controlled trial. Int. Orthop..

[42] Zhang S (2017). The effects of multiple intravenous tranexamic acid administrations after total knee arthroplasty on fibrinolytic activity and inflammatory response. Chin. J. Orthop..

[43] Zheng H (2019). Effect of local application of tranexamic acid on coagulation and fibrinolysis in patients undergoing spinal surgery. Med. J. Chin. PLA.

[44] Seol YJ (2016). Effect of tranexamic acid on blood loss and blood transfusion reduction after total knee arthroplasty. Knee Surg. Relat. Res..

[45] Zhen Z (2016). Research progress in the application of tranexamic acid in spinal surgery. J. Pract. Orthop..

[46] Farrokhi MR (2011). Efficacy of prophylactic low dose of tranexamic acid in spinal fixation surgery: A randomized clinical trial. J. Neurosurg. Anesthesiol..

[47] Ji Q (2016). Two ways of medication of tranexamic acid: A comparison between the two methods regarding perioperative blood loss and safety in patients undergoing lumbar surgery. Med. J. Qilu.

[48] Liu Y (2019). Effect of Tranexamic Acid in Reducing Perioperative Bleeding of the Elderly Undergoing Posterior Lumbar Surgery of 3 Segments and the Safety. Geriatr. Health Care.

[49] Gu L-J (2018). A case report of tranexamic acid associated epilepsy in spine surgery. Chin. J. Orthop. Trauma.

